# Rare Copy Number Variations and Predictors in Children With Intellectual Disability and Epilepsy

**DOI:** 10.3389/fneur.2018.00947

**Published:** 2018-11-19

**Authors:** Miriam Kessi, Juan Xiong, Liwen Wu, Lifen Yang, Fang He, Chen Chen, Nan Pang, Haolin Duan, Wen Zhang, Ahmed Arafat, Fei Yin, Jing Peng

**Affiliations:** ^1^Department of Pediatrics, Xiangya Hospital, Central South University, Changsha, China; ^2^Hunan Intellectual and Developmental Disabilities Research Center, Changsha, China

**Keywords:** intellectual disability, global developmental delay and epilepsy, copy number variations, independent predictors, clinical markers

## Abstract

**Introduction:** The concurrence of intellectual disability/global developmental delay and epilepsy (ID/GDD-EP) is very common in the pediatric population. The etiologies for both conditions are complex and largely unknown. The predictors of significant copy number variations (CNVs) are known for the cases with ID/GDD, but unknown for those with exclusive ID/GDD-EP. Importantly, the known predictors are largely from the same ethnic group; hence, they lack replication.

**Purpose:** We aimed to determine and investigate the diagnostic yield of CNV tests, new causative CNVs, and the independent predictors of significant CNVs in Chinese children with unexplained ID/GDD-EP.

**Materials and methods:** A total of 100 pediatric patients with unexplained ID/GDD-EP and 1,000 healthy controls were recruited. The American College of Medical Genetics guideline was used to classify the CNVs. Additionally, clinical information was collected and compared between those with significant and non-significant CNVs.

**Results:** Twenty-eight percent of the patients had significant CNVs, 16% had variants of unknown significance, and 56% had non-significant CNVs. In total, 31 CNVs were identified in 28% (28/100) of cases: 25 pathogenic and 6 likely pathogenic. Eighteen known syndromes were diagnosed in 17 cases. Thirteen rare CNVs (8 novel and 5 reported in literature) were identified, of which three spanned dosage-sensitive genes: 19q13.2 deletion (*ATP1A3*), Xp11.4-p11.3 deletion (*CASK*), and 6q25.3-q25.3 deletion (*ARID1B*). By comparing clinical features in patients with significant CNVs against those with non-significant CNVs, a statistically significant association was found between the presence of significant CNVs and speech and language delay for those aged above 2 years and for those with facial malformations, microcephaly, congenital heart disease, fair skin, eye malformations, and mega cisterna magna. Multivariate logistic regression analysis allowed the identification of two independent significant CNV predictors, which are eye malformations and facial malformations.

**Conclusion:** Our study supports the performance of CNV tests in pediatric patients with unexplained ID/GDD-EP, as there is high diagnostic yield, which informs genetic counseling. It adds 13 rare CNVs (8 novel), which can be accountable for both conditions. Moreover, congenital eye and facial malformations are clinical markers that can aid clinicians to understand which patients can benefit from the CNV testing and which will not, thus helping patients to avoid unnecessary and expensive tests.

## Introduction

Intellectual disability (ID) is a complex neurodevelopmental ailment characterized by low intelligence quotient (IQ < 70) and restrictions in adaptive functioning, normally diagnosed before the age of 18 years (1). Global developmental delay (GDD) is defined as significant delay in two or more developmental domains. Significant delay is determined by performance that is two or more standard deviations lower than the mean on objective, norm-referenced, and age-appropriate testing in two or more domains (2). The term GDD is used for young children who are < 5 years of age, as there is some disagreement on how to objectively measure IQ and cognition in a consistent, reliable, and valid fashion in these patients (3). Epilepsy (EP) is a brain disease characterized by two unprovoked seizures >24 h apart (International League Against Epilepsy [ILAE]) (4). Epilepsy is a very common disease in children with intellectual disability/global developmental delay (ID/GDD) with an estimated prevalence of about 22.2% (5, 6). Patients with both intellectual disability/global developmental delay and epilepsy (ID/GDD-EP) have a mortality rate 3.3 times higher than those with only intellectual disability/global developmental delay (7).

Genetic etiologies, prenatal illnesses, postnatal illnesses, environmental factors, and metabolic diseases can account for ID/GDD-EP. For a substantial proportion of people with both conditions, the underlying etiology has yet to be elucidated. Camfield et al. found in their study that ~37% of their patients had unknown etiology (8). These patients without obvious cause can be classified as patients with unexplained ID/GDD-EP. The obvious causes include conditions such as metabolic diseases, infectious diseases, immunological conditions, autoimmune diseases, brain injuries, Down syndrome, and tuberous sclerosis complex.

Genetic factors play a major role in etiology, especially in pediatric patients, who are highly heterogeneous (8). Copy number variations (CNVs) are important mechanisms of genomic diversity and evolutionary changes in humans. Over the past decade, genomewide identification of CNVs has become efficient, allowing the detection of causative submicroscopic chromosomal aberrations, especially in neuropsychiatric disorders, including autism spectrum disorder, ID/GDD, EP, and schizophrenia (9). Borlot et al. found a high prevalence of pathogenic CNVs in their cohort of adults with pediatric-onset epilepsy and intellectual disability (10). However, only a few studies have focused on the identification of the role of CNV tests in pediatric patients with ID/GDD-EP, especially from China.

Copy number variations can be detected by multiple methods, including array-based comparative genomic hybridization (aCGH), single nucleotide polymorphism (SNP) arrays, and next-generation sequencing (CNV seq) (11). The CNV tests are expensive despite the fact that they are useful in identifying the etiologies of neuropsychiatric disorders. The diagnostic yield ranges from 15 to 20%; hence, not all cases have pathogenic CNVs (11). Therefore, it is important to have clinical markers/predictors that could guide clinicians in ordering this particular test. There are few studies that focused on identifying the predictors of significant (pathogenic or likely pathogenic) CNVs in patients with ID/GDD, and most of them are from the same ethnic group and, hence, lack replication (12–15). Moreover, no study has focused on identifying the predictors of significant CNVs among patients with exclusive unexplained ID/GDD-EP.

In this study, we sought to determine and investigate the diagnostic yield of CNV tests, new causative CNVs, and the independent predictors of significant CNVs in Chinese children with unexplained ID/GDD-EP. This study identifies new CNVs, which can be accountable for both ID/GDD and EP, and provides some clinical markers to help clinicians understand which patients can benefit from the CNV testing and which will not, thus helping patients avoid unnecessary and expensive tests, especially for resource-limited countries.

## Materials and methods

### Ethical clearance

This study was reviewed and approved by the Institutional Ethics Committee of Xiangya Hospital Central South University, thus complying with the treaty agreed to in 1964 in Helsinki by the World Medical Association on ethical principles of human research for medical purposes and subsequent revisions of the same (2013). Both informed and written consent were obtained from the subjects.

### Human subjects

A total of 100 pediatric patients, diagnosed by senior neurologists to have unexplained ID/GDD-EP at the Children's Intellectual Disability Medical Institution, Department of Pediatric Neurology, Xiangya Hospital, were recruited from the hospital database retrospectively. In total, 1,000 in-house healthy controls from the Chinese population were recruited. Inclusion criteria for this cross-sectional study include: (1) aged 14 years and below, (2) diagnosed with unexplained ID with IQ < 70 or with unexplained GDD with DQ < 85, (3) diagnosed with unexplained EP, which happened after ID/GDD, and (4) underwent a CNV test. The exclusion criteria include: (1) diagnosed with only unexplained ID/GDD without EP, (2) diagnosed with only unexplained EP without ID/GDD, (3) diagnosed with ID/GDD that happened after EP, and (4) diagnosed with unexplained ID/GDD-EP, but did not undergo a CNV test.

### Diagnostic protocol

The assessment of the ID was done according to diagnostic criteria of the DSM-5 for intellectual disabilities (Diagnostic and Statistical Manual of Mental Disorders, Fifth Edition, American Psychiatric Association, 2013). Observations, clinical interview, and standardized age-related rating scales were used for the assessment of the adaptive functioning. However, the diagnosis was often initially formulated based on clinical judgment, rather than on formal standardized assessments, especially for young patients (16). Standardized age-related rating scales that were used include: Gesell Developmental Schedules for patients younger than 2–4 years, Wechsler Preschool and Primary Scale of Intelligence-Fourth Edition (WPPSI-IV) for patients between 4 and 6 years, and Wechsler Intelligence Scale for Children-Fourth Edition (WISC-IV) for patients who were 6 years old or above. Clinical judgment was utilized to grade the severity of GDD for the patients aged below 2 years. Patients with deficits in their adaptive and intellectual functioning with an onset during their developmental period were classified into four categories, such as mild ID when their IQ values ranged from 55 to 70, moderate ID when IQ values ranged from 40 to 55, severe ID when IQ values ranged from 25 to 40, and profound ID when IQ values were less than 25. Patients with DQ ranging from 65 to 84 were considered to have mild GDD, those with DQ ranging from 45 to 64 were considered to have moderate GDD, and those with DQ of 44 or less were considered to have severe GDD. Epilepsy was diagnosed according to International League Against Epilepsy (ILAE) guidelines. Clinical, neurophysiological, and imaging data were considered in defining phenotypes. Phenotypes were classified into known electro-clinical syndromes according to the ILAE classification, where possible, or into “unclassified epilepsy” when they did not fit the criteria for syndromes.

### Copy number variation tests

Whole-genome CNVs were detected using the following: CNV_01 Affymetrix aGGH+SNP Microarray for 8 patients, Illumina HumanCytoSNP-12 BeadChip for 6 patients, and low-depth whole-genome sequencing for the remaining patients. One-thousand healthy controls were genotyped using the Illumina Human610-Quad BeadChip (17). BeadChip the CNV_01 Affymetrix aGGH+SNP Microarray incorporates 200,000 of the best SNPs for CNV testing (https://www.thermofisher.com). The Illumina HumanCyto-SNP12 BeadChip is a powerful, whole-genome scanning panel that incorporates 300,000 of the best SNPs for CNV testing and has dense coverage of around 250 genomic regions commonly screened in cytogenetics laboratories (https://www.illumina.com). Low-depth whole-genome sequencing is a cost-effective approach, which detects low frequency and rare variation in complex trait-association studies (http://www.biorxiv.org). Chromosome coordinates refer to chromosome build (hg19).

### Copy number variation analysis and classification

The pathogenicity of CNVs was predicted based on their size and contained gene according to the American College of Medical Genetics (ACMG) guidelines for interpretation of postnatal CNVs (18). All detected CNVs in patients were, firstly, compared with the DGV database (Database of Genomic Variants, http://dgv.tcag.ca/gb2/gbrowse/dgv) and 1,000 healthy controls (17). The identified candidate CNVs, which did not overlap with those in the DGV and healthy controls, were then compared with DECIPHER (Database of Chromosomal Imbalance and Phenotype in Humans using Ensemble Resources, https://decipher.sanger.ac.uk) and ClinVar (https://www.ncbi.nlm.nih.gov/clinvar). The genes involved in the chromosomal region of interest and their functions were studied for their potential role in ID/GDD or EP/both by using all available evidence in databases such as the Online Mendelian Inheritance in Man (OMIM), ClinGen, Gene Reviews, and PubMed. For each relevant OMIM gene that could play a role in the patient's phenotype, the residual variation intolerance score (RVIS) and the percentile of most intolerant genes (MIG) to guide the interpretation of potential clinical significance were applied (19). The RVIS is based on allele frequency and represented in whole-exome sequence data from the National Heart, Lung, and Blood Institute Exome Sequencing Project 6500 datasets. A gene with a positive score has more common functional variation and a gene with a negative score has less variation and is referred to as intolerant. The more intolerant a gene is, the more likely it is associated with the disease, if mutated or at abnormal levels. Additionally, the Human Protein Atlas was used to study their tissue expression (https://www.proteinatlas.org/). Parents were analyzed, where available, to determine if the CNV had arisen *de novo* or was inherited. Furthermore, parental phenotypes were taken into account to help determine if the CNV was significant.

### Validation of suspected pathogenic copy number variations

Parents of patients with suspected pathogenic CNVs were tested for validation whenever available and gave consent. The fluorescence *in situ* hybridization or quantitative polymerase chain reaction or CNV tests were used for validation and identification of the mode of CNV inheritance.

### Statistical analysis

For the purpose of analysis, we divided patients into two major groups. The first group consisted of patients with significant CNVs (pathogenic and likely pathogenic). The second group consisted of patients with non-significant CNVs (likely benign, benign, or no CNV detected). Carriers of variants of unknown significance (VOUS) were excluded from the analysis, since the pathogenic role was unknown. The phenotypes of the two groups were compared and analyzed using IBM SPSS Statistics 22.0 software (IBM, Armonk, NY, USA). We excluded the missing values in data analysis. We compared the clinical data of patients with significant CNVs and those with non-significant CNVs. The association between each clinical feature and CNV test results was first established using a Chi-squared test (or Fisher's exact test when appropriate). Fisher's exact test (2-sided), rather than a Chi-squared test (2-sided), was used when one or more of the cells had an expected value of < 5. Furthermore, to identify the independent predictors of significant CNVs, all significant variables in the univariate analysis were subjected into a multivariate logistic regression model and backward stepwise selection of variables was performed, using the presence of significant CNVs as a dependent dichotomous variable. Odds ratios, standard error, 95% confidence intervals (CI), and positive and negative predictive values were calculated. Results with a value of *P* ≤ 0.05 were considered to be statistically significant.

## Results

### Cohort characteristics

Our study consisted of 100 patients with unexplained ID/GDD-EP, where 63% (63/100) were males and 37% (37/100) were females. The mean age of diagnosis of EP and ID/GDD was 30.45 months ± 33.2038 SD and 9.8 months ± 13.8082 SD, respectively. About 7% (7/100) had mild ID/GDD, 36% (36/100) had moderate ID/GDD, and 57% (57/100) had severe ID/GDD. All patients underwent brain imaging (MRI or CT scan) and EEG examination. Moreover, 47 patients had brain abnormalities such as ventriculomegaly, hippocampal sclerosis, mega cisterna magna, arachnoid cysts, hydrocephalus, widened subarachnoid space, white matter dysplasia, brain atrophy, and smaller pituitary gland, while 53 patients had normal findings. About 94% (94/100) of the patients had abnormal EEG findings, while 6% (6/100) had normal findings (Table 1).

**Table 1:** Cohort characteristics of the 100 patients with both unexplained intellectual disability/global developmental delay and epilepsy.

**Clinical characteristics**	**Sample size (*N*) and percentage**
**SEX**	
Male	63.0% (63/100)
Female	37.0% (37/100)
**DIAGNOSIS AGE**	
Mean age of diagnosis of epilepsy	30.450 months ± 33.2038SD. Range = 0–155 months
Mean age of diagnosis of intellectual disability/global developmental delay.	9.8 months ± 13.8082SD. Range = 1–85 months.
**INTELLECTUAL DISABILITY CATEGORIES**	
Mild	7.0% (7/100)
Moderate	36.0% (36/100)
Severe	57.0% (57/100)
**MRI/CT SCAN RESULTS**	
Normal	53.0% (52/100)
Abnormal	47.0% (47/100)
**EEG RESULTS**	
Normal	6.0% (6/100)
Abnormal	94.0% (94/100)

*MRI, magnetic resonance imaging; CT, computed tomography; EEG, electroencephalogram*.

### Copy number variation test results

Twenty-four percent (24/100 cases) had at least one pathogenic CNV each, 4% (4/100 cases) had at least one likely pathogenic CNV, 16% (16/100 cases) had VOUS, 49% (49/100 cases) had benign CNVs, and 7% (7/100 cases) had no CNV detected. Thirty-one CNVs (25 pathogenic and 6 likely pathogenic) were identified in 28% (28/100 cases) as possible causes for unexplained ID/GDD-EP. One case had 3 CNVs (all were likely pathogenic), one case had 2 pathogenic CNVs, 23 cases had one pathogenic CNV each, and 3 cases had one likely pathogenic CNV each. The size of their CNVs ranged from 0.11 Mb to 21 Mb. Twenty of all detected pathogenic or likely pathogenic CNVs were deletions and 13 were duplications. Eight patients had *de novo* CNVs, while others had an unknown mode of inheritance since their parents were not tested (Supplementary Table 1).

### Identified syndromes

Eighteen known syndromes were diagnosed in 17 cases as follows: Angelman syndrome for two cases (case number 3 and 23), Prader-Willi syndrome for two cases (case number 8 and 15), Lubs X-linked mental retardation syndrome for three cases (case number 13, 16, and 21), 16p11.2-p12.2 microdeletion syndrome for three cases (case number 9, 18, and 25), 1p36 microdeletion syndrome for two cases (case number 17 and 26), 2q33.1 deletion syndrome for one case (case number 27), 17p13.1 microdeletion syndrome for one case (case number 2), 1q21.1 recurrent microdeletion syndrome for one case (case number 5), 3q29 microduplication syndrome and 1q43q44 microdeletion syndrome for one case (case number 20), and Wolf-Hirschhorn syndrome for one case (case number 28). One case had two CNVs that present two syndromes: 3q29 microduplication syndrome and 1q43q44 microdeletion syndrome. This information is summarized in Table 2.

**Table 2:** Eighteen identified syndromes.

**Case No**	**Seizure type**	**ID/GDD category**	**CNV**	**Genomic coordinates (NCBI 37/hg19)**	**Associated genetic syndrome**
2	Tonic	Severe	Unknown inheritance 17p13.1-p13.1 del (380 Kb)	Chr17: 6861627–7241627	17p13.1 microdeletion syndrome (OMIM 613776)
5	Focal	Severe	*De novo* 1q21.1-q21.2 del (3.99 Mb)	Chr1: 143896003–147888300	1q21.1 recurrent microdeletion syndrome (OMIM 612474)
7	Myoclonus	Severe	Unknown inheritance 15q11.2-q13.1 del (4.92 Mb)	Chr15: 23609854–28526207	Angelman syndrome (OMIM 105830)
8	Focal	Moderate	Unknown inheritance 15q11.2-q13.1 del (4.93 Mb)	Chr15: 23609854–28536207	Prader-Will syndrome (OMIM 176270)
9	Focal	Severe	Unknown inheritance 16p11.2-p11.2 del (550 Kb)	Chr16: 29661922–30211922	16p11.2-p12.2 microdeletion syndrome (OMIM 613604)
13	Tonic-clonic	Severe	Unknown inheritance Xq28-q28 dup (340 Kb)	ChrX: 153232100–153572100	Lubs X-linked mental retardation syndrome (OMIM 300260)
15	Tonic	Severe	Unknown inheritance 15q11.2-q13.1 del (5.15 Mb)	Chr15: 23609854–28756207	Prader-Willi syndrome (OMIM 176270)
16	Tonic	Moderate	Unknown inheritance Xq28-q28 dup (210 Kb)	ChrX: 153362100–153572100	Lubs X-linked mental retardation syndrome (OMIM 300260)
17	Focal, spasm	Severe	Unknown inheritance 1p36.33-p36.33 del (1.10 Mb)	Chr1: 746369–1846369	1p36 microdeletion syndrome (OMIM 607872)
18	Focal	Moderate	Unknown inheritance 16p11.2-p11.2 del (640 Kb)	Chr16: 29571922–30211922	16p11.2-p12.2 microdeletion syndrome (OMIM 613604)
20	Focal	Severe	*De novo* 1q44q44 del (4.6Mb)	Chr1: 244307212–248903211	1q43q44 microdeletion syndrome(OMIM 612337)
			*De novo* 3q29-q29 dup (880Kb)	Chr3: 195732252–196612252	3q29 microduplication syndrome (OMIM 611936)
21	Tonic	Moderate	Unknown inheritance Xq28-q28 dup (210 Kb)	ChrX: 153362100– 153572100	Lubs X-linked mental retardation syndrome (OMIM 300260)
23	Tonic	Severe	Unknown inheritance 15q11.2-q13.1 del (6.01 Mb)	Chr15: 22751194–28756207	Angelman syndrome (OMIM 105830)
25	Tonic	Moderate	*De novo* 16p11.2-p11.2 del (640 Kb)	Chr16: 29571922–30211922	16p11.2-p12.2 microdeletion syndrome (OMIM 613604)
26	Tonic	Moderate	Unknown inheritance 1p36.32-p36.23 del (5.3 Mb)	Chr1: 3417960–8774451	1p36 microdeletion syndrome (OMIM 607872)
27	Focal	Severe	*De novo* 2q33.1-q34 del (8.9 Mb)	Chr2: 202781244–211695915	2q33.1 deletion syndrome/Glass syndrome (OMIM 612313)
28	Focal	Severe	Unknown inheritance 4p16.3-p16.1 del (7.3 Mb)	Chr4: 68345–7109830	Wolf-Hirschhorn syndrome (OMIM 194190)

*M, male; F, female; Del, deletion; Dup, duplication; ID, intellectual disability; GDD, global developmental delay; EP, epilepsy; OMIM, Online Mendelian Inheritance in Man*.

### Rare pathogenic CNVs and their candidate genes

Seven rare pathogenic CNVs were identified, and three of them span dosage-sensitive genes: deletion at 19q13.2 (*ATP1A3*) for case number 1, Xp11.4-p11.3 (*CASK*) for case number 4, and 6q25.3-q25.3 (*ARID1B*) for case number 6. Three CNVs were large deletions, meaning >3 Mb and one is *de novo* duplication of 14.82 Mb. This information is summarized in Table 3.

**Table 3:** Seven rare pathogenic CNVs and their candidate genes information.

**Case number/Sex/ CNV**	**Genes in the region**	**Known or candidate gene (s) for ID/GDD or EP/both**	**Associated diseases and mode of inheritance**	**RVIS (% of MIG)**	**Gene expression studies**	**CNV Reported in literature**	**Classification**
1/M *De novo* 19q13.2 deletion (333 Kb)	11 RefSeq genes including *ATP1A3, ERF*	*ATP1A3*	Alternating hemiplegia of childhood 2 (OMIM 614820) (AD)	−1.53 (3.37%)	High expression in neural tissues	Yes	P
3/F Unknown inheritance 10q11.23-q22.1 deletion (21 Mb)	154 RefSeq genes including *ANK3*.	*ANK3*	Mental retardation, autosomal recessive, 37(OMIM 615493) (AR)	−3.5 (0.33%)	Medium expression in neural tissues.	No	P
4/F Unknown inheritance Xp11.4-p11.3 deletion (2.14 Mb)	15 RefSeq genes including *CASK*	*CASK*	Mental retardation and microcephaly with pontine and cerebellar hypoplasia(MICPCH) (OMIM 300749) (XLD): Mental retardation, with or without nystagmus (OMIM 300422) (XLD): FG syndrome 4 (OMIM 300422) (XLD).	−0.715 (14.4%)	Medium expression in neural tissues	No	P
6/F Unknown inheritance 6q25.3-q25.3 del (240 Kb)	2 RefSeq genes including *ARID1B*	*ARID1B*	Coffin-Siris syndrome 1(OMIM 135900) (AD)	−2.62 (0.8%)	High expression in neural Tissues	Yes	P
10/M *De novo* 10q23.31-q24.33 duplication (14.82Mb)	245 RefSeq genes, including *C10orf2, CNNM2, and LGI1*.	*C10orf2, CNNM2, LGI1*	Mitochondrial DNA depletion syndrome 7 (hepatocerebral type) (OMIM 271245) (AR): Hypomagnesemia, seizures, and mental retardation. (OMIM 616418) (AR,AD): Epilepsy, familial temporal lobe, 1 (OMIM 600512) (AD).	−0.78 (12.97%) −0.98 (8.75%) −0.71 (14.4%)	High expression in neural tissues	No	P
22/M Unknown inheritance 8p21.2-p21 del (10.8Mb)	106 RefSeq genes including *CHRNA2*	*CHRNA2*	Epilepsy, nocturnal frontal lobe, type 4 (OMIM 610353) (AD).	−0.753 (13.67%)	Low expression in neural tissues	Yes	P
24/M *De novo* 8p23.3-p23.2 deletion (5.57 Mb)	20 RefSeq genes including *CLN8*	*CLN8*	Ceroid lipofuscinosis, neuronal, 8, Northern epilepsy variant (OMIM 610003) (AR).	−0.14 (43.77%)	Medium expression in neural tissues	Yes	P

*M, male; F, female; AD, autosomal dominant; AR, autosomal recessive; CNV, copy number variation; ID, intellectual disability; GDD, global developmental delay; EP, epilepsy; MIG, most intolerant genes; RefSeq, reference sequence; RVIS, Residual Variation Intolerance Score; OMIM, Online Mendelian Inheritance in Man; MRI, magnetic resonance imaging; NIL, nothing*.

### Rare likely pathogenic CNVs

Six rare likely pathogenic CNVs were identified. They span genes whose function is unclear, but correlate with patients' phenotypes. These include duplication at 4q24-q24 (*CENPE*) for two cases, 10q21.2-q21.2 (*ANK3*), 14q32.31-q32.31 (*DYNC1H1*), 1p34.2-p34 (*SZT2*), and Xp21.3-p21.3 (*IL1RAPL1*). This information is summarized in Table 4.

**Table 4:** Six rare likely pathogenic CNVs and their candidate genes information.

**Case number/Sex/ CNV**	**Genes in the region**	**Known or candidate gene (s) for ID/GDD or EP/both**	**Associated diseases and mode of inheritance**	**RVIS (% of MIG)**	**Gene expression studies**	**CNV reported in literature**	**Classification**
11/M *De novo* 4q24-q24 duplication (150 Kb)	2 RefSeq genes including *CENPE*	*CENPE*	Microcephaly 13, primary, autosomal recessive(OMIM 616051) (AR)	−0.55 (20%)	Low expression in neural tissues	No	LP
12/M Unknown inheritance 1p34.2-p34 duplication (160Kb)	7 RefSeq genes including *SZT2*	*SZT2*	Epileptic encephalopathy, early infantile, 18(OMIM 616418)(AR)		High expression in neural tissues	No	LP
12/M Unknown inheritance 4q24-q24 duplication (170 Kb)	3 RefSeq genes including *CENPE*	*CENPE*	Microcephaly 13, primary, autosomal recessive(OMIM 616051) (AR)	−0.55 (20%)	Low expression in neural tissues	No	LP
12/M Unknown inheritance 10q21.2-q21.2 duplication (180 Kb)	*ANK3*	*ANK3*(part)	Mental retardation, autosomal recessive, 37(OMIM 615493) (AR)	−3.5 (0.334%)	Medium expression in neural tissues	No	LP
14/M Unknown inheritance Xp21.3-p21.3 duplication (110 Kb)	2RefSeq genes including *IL1RAPL1*	*IL1RAPL1*	Mental retardation, X-linked 21/34 (OMIM 300143) (XLR)	−0.60 17.74%	Highly expression in neural tissues	Yes	LP
19/F Unknown inheritance 14q32.31-q32.31 duplication (140 Kb)	2 RefSq genes including DYNC1H1	*DYNC1H1*	Mental retardation, autosomal dominant 13(OMIM 614563) (AD)	−6.01 (0.04%)	Highly expression in neural tissues	No	LP

*M, male; F, female; AD, autosomal dominant; AR, autosomal recessive; CNV, copy number variation; ID, intellectual disability; GDD, global developmental delay; EP, epilepsy; MIG, most intolerant genes; RefSeq, reference sequence; RVIS, Residual Variation Intolerance Score; OMIM, Online Mendelian Inheritance in Man; MRI, magnetic resonance imaging; NIL, nothing*.

### Comparison of CNVs and clinical findings (*N* = 84)

By comparing seizure characteristics, clinical information, EEG findings, brain imaging findings, and clinical features in patients with significant CNVs against those with non-significant CNVs, a statistically significant association was found between the presence of significant CNVs and speech and language delay for those aged above 2 years (*P* = 0.025) and for those with facial malformations (*P* = 0.000), microcephaly (*P* = 0.014), congenital heart disease (*P* = 0.005), fair skin (*P* = 0.034), eye malformations (*P* = 0.037), and mega cisterna magna (*P* = 0.034) (Table 5). Eye malformations (*P* = 0.032) and facial malformations (*P* = 0.027) were identified as independent predictors of significant CNVs according to multivariate logistic regression analysis (Table 6).The positive predictive value for the adjusted model was 65%, whereas negative predictive value was 80% (Table 7).

**Table 5:** Comparison of CNVs and clinical features of patients with both unexplained intellectual disability/global developmental delay and epilepsy.

**Clinical feature**	**Copy number variation results**		**Overall (*N* = 84)**	***P*-value**
	**Pathogenic (*n* = 28)**	**Benign (*n* = 56)**	
Onset of seizures at 30 months and above	46.4% (13/28)	32.1% (18/56)	36.9% (31/84)	0.201
Intellectual disability onset at 3.5 months and above	35.7% (10/28)	55.4% (31/56)	48.8% (41/84)	0.090
Age of mother 35 years and above	11.1% (3/27)	9.3% (5/54)	9.9% (8/81)	0.792
Age of father 35 years and above	11.1% (3/27)	14.0% (7/50)	13.0% (10/77)	1.000
Birth weight < 2,500 g	17.9% (5/28)	7.4% (4/54)	11.0% (9/82)	0.262
Birth weight > 4,000 g	7.1% (2/28)	5.5% (3/55)	6.0% (5/84)	1.000
Prematurity < 37 weeks GA	0.0% (0/28)	7.3% (4/55)	4.8% (4/83)	0.295
Intrauterine growth restriction	17.9% (5/28)	5.6% (3/54)	9.8% (8/82)	0.115
Hypoxia	21.4% (6/28)	20.0% (11/55)	20.5% (17/83)	0.879
Consanguineous family	0.0% (0/28)	1.8% (1/55)	1.2% (1/83)	1.000
Prenatal complications	46.4% (13/28)	36.4% (20/55)	39.8% (33/83)	0.376
Family history of EP/ID/both	35.7% (10/28)	38.2% (21/55)	37.3% (31/83)	0.826
Past history of other illnesses prior to diagnosis of both EP and ID	64.3% (18/28)	50.0% (28/56)	54.8% (46/84)	0.215
Primigravida mother	28.6% (8/28)	32.7% (18/55)	31.3% (26/83)	0.700
Speech and language delay >2 years	70.8% (17/24)	43.1% (22/51)	52.0% (39/75)	0.025
Microcephaly	42.9% (12/28)	17.9% (10/56)	26.2% (22/84)	0.014
Macrocephaly	3.6% (1/28)	0.0% (0/56)	1.2% (1/84)	0.333
Facial malformations	75.0% (21/28)	33.9% (19/56)	47.6% (40/84)	0.000
Skeletal malformations	17.9% (5/28)	8.9% (5/56)	11.9% (10/84)	0.234
Brachydactyly	3.6% (1/28)	0.0% (0/56)	1.2% (1/84)	0.333
Depigmented skin spots	7.1% (2/28)	7.1% (4/56)	7.1% (6/84)	1.000
Fair skin	10.7% (3/28)	0.0% (0/56)	3.6% (3/84)	0.034
Café au lait spots	3.6% (1/28)	10.7% (6/56)	8.3% (7/84)	0.416
Hearing impairment	10.7% (3/28)	7.1% (4/56)	8.3% (7/84)	0.681
Ear malformations	10.7% (3/28)	10.7% (6/56)	10.7% (9/84)	1.000
Eye malformations (impairment, strabismus and nystagmus)	25.0% (7/28)	7.1% (4/56)	13.1% (11/84)	0.037
Congenital heart disease	28.6% (8/28)	5.4% (3/56)	13.1% (11/84)	0.005
Genital malformations	10.7% (3/28)	8.9% (5/56)	9.5% (8/84)	1.000
Umbilical hernia	0.0% (0/28)	1.8% (1/56)	1.2% (1/84)	1.000
Hypotonia	7.1% (2/28)	7.1% (4/56)	7.1% (6/84)	1.000
Hypertonia	7.1% (2/28)	5.4% (3/56)	6.0% (5/84)	1.000
Hyperreflexia	7.1% (2/28)	5.4% (3/56)	6.0% (5/84)	1.000
Hyporeflexia	3.6% (1/28)	8.9% (5/56)	7.1% (6/84)	0.658
Abnormal power/strength	7.1% (2/28)	3.6% (2/56)	4.8% (4/84)	0.598
Positive Babinski sign	3.6% (1/28)	7.1% (4/56)	6.0% (5/84)	0.661
Positive clonus test	0.0% (0/28)	1.8% (1/56)	1.2% (1/84)	1.000
Abnormal behaviors	46.4% (13/28)	30.4% (17/56)	35.7% (30/84)	0.147
**CHARACTER OF SEIZURES**				
Tonic seizure	57.1% (16/28)	50.0% (28/56)	52.4% (44/84)	0.537
Myoclonic seizure	7.1% (2/28)	3.6% (2/56)	4.8% (4/84)	0.598
Spasm seizure	10.7% (3/28)	3.6% (2/56)	6.0% (5/84)	0.327
Partial seizure	39.3% (11/28)	37.5% (21/56)	38.1% (32/84)	0.834
Clonic seizure	3.6% (1/28)	0.0% (0/56)	1.2% (1/84)	0.333
Seizure controlled with one AED	35.7% (10/28)	41.1% (23/56)	39.3% (33/84)	0.636
Seizure controlled by two AED	14.3% (4/28)	5.4% (3/56)	8.3% (7/84)	0.215
Fever-related	28.6% (8/28)	18.2% (10/55)	21.7% (18/83)	0.278
Seizure control after 3 months	50.0% (14/28)	50.0% (28/56)	50.0% (42/84)	1.000
**EEG FINDINGS**				
Abnormal EEG	92.9% (26/28)	94.6% (53/56)	94.0% (79/84)	0.744
Hypsarrhythmia	3.6% (1/28)	5.4% (3/56)	4.8% (4/84)	1.000
Focal discharge	25.0% (7/28)	14.3% (8/56)	17.9% (15/84)	0.227
Sharp wave	32.1% (9/28)	28.6% (16/56)	29.8% (25/84)	0.736
Spike wave	46.4% (13/28)	46.4% (26/56)	46.4% (39/84)	1.000
Slow waves	14.3% (4/28)	19.6% (11/56)	17.9% (15/84)	0.764
Continuous epileptic activity	7.1% (2/28)	14.3% (8/56)	11.9% (10/84)	0.484
Right hemisphere origin	3.6% (1/28)	12.5% (7/56)	9.5% (8/84)	0.259
Left hemisphere origin	7.1% (2/28)	17.9% (10/56)	14.3% (12/84)	0.321
Both hemisphere origin	82.1% (23/28)	62.5% (35/56)	69.0% (58/84)	0.066
Sleep stage	35.7% (10/28)	39.3% (22/56)	38.1% (32/84)	0.751
Awake stage	3.6% (1/28)	14.3% (8/56)	10.7% (9/84)	0.260
Both sleeping stages	53.6% (15/28)	39.3% (22/56)	44.0% (37/84)	0.214
Occipital lobe origin	10.7% (3/28)	10.7% (6/56)	10.7% (9/84)	1.000
Frontal lobe origin	42.9% (12/28)	41.1% (23/56)	41.7% (35/84)	0.876
Temporal lobe origin	21.4% (6/28)	14.3% (8/56)	16.7% (14/84)	0.408
Frontal central region	17.9% (5/28)	12.5% (7/56)	14.3% (12/84)	0.508
Central temporal region	7.1% (2/28)	16.1% (9/56)	13.1% (11/84)	0.322
**MRI/CT SCAN FINDINGS**				
Abnormal MRI/CT scan	50.0% (14/28)	46.4% (26/56)	47.6% (40/84)	0.757
Brain atrophy	14.3% (4/28)	5.4% (3/56)	8.3% (7/84)	0.215
Mega cisterna magna	10.7% (3/28)	0.0% (0/56)	3.6% (3/84)	0.034
Corpus callosum dysplasia	3.6% (1/28)	1.8% (1/56)	2.4% (2/84)	1.000
Hydrocephalus	3.6% (1/28)	0.0% (0/56)	1.2% (1/84)	0.333
White matter dysplasia	3.6% (1/28)	7.1% (4/56)	6.0% (5/84)	0.661
Arachnoid cyst	3.6% (1/28)	1.8% (1/56)	2.4% (2/84)	1.000
Hippocampal sclerosis	3.6% (1/28)	1.8% (1/56)	2.4% (2/84)	1.000
**SEVERITY OF ID/GDD**				
Severe	53.6% (15/28)	57.1% (32/56)	56% (47/84)	0.756
Moderate	42.9% (12/28)	33.9%(19/56)	36.9%(31/84)	0.424
Mild	3.6% (1/28)	8.9% (5/56)	7.1% (6/84)	0.656

*MRI, magnetic resonance imaging; CT, computed tomography; EEG, electroencephalogram; AED, antiepileptic drugs; ID, intellectual disability; GDD, global developmental delay*.

**Table 6:** Results of the multivariate logistic regression analysis for the clinical features strongly associated with significant CNVs, univariate analysis.

**Independent predictors**	***P*-value**	**OR**	**Standard error**	**OR 95% CI**
Speech delay >2 years	0.261	2.005	0.619	0.596–6.746
Facial malformations	0.027	4.051	0.633	1.172–14.005
Congenital heart disease	0.172	3.261	0.866	0.597–17.808
Eye malformations	0.032	6.012	0.838	1.163–31.085
Microcephaly	0.410	1.720	0.659	0.473–6.259

*CNVs, copy number variations; OR, Odds ratio; CI, confidence interval*.

**Table 7:** Results of the assessment of predictive value of the multivariate model.

**Observed**	**Predicted pathogenic/benign CNVs**		**Percentage of correct predictions**
	**Pathogenic**	**Benign**
Pathogenic	13	11	54.2
Benign	7	44	86.3
Overall predicted correct percentage			76

*Sensitivity = 13/(13+11)% = 54.2%; Specificity = 44/(7+44)% = 86.3%; False positive = 7/(7+13)% = 35%; False negative = 11/(11+44)% = 20%; Positive predictive value = 13/(13+7)% = 65%; Negative predictive value = 44/(44+11)% = 80%*.

*Using this model, the probability of a patient having pathogenic CNVs when they have above clinical features is 65%*.

*Using this model, the probability of a patient having benign CNVs when they don‘t have above clinical features is 80%*.

## Discussion

This study focuses on pediatric patients with unexplained ID/GDD-EP, with its major aims being to identify the following: the diagnostic yield of CNV tests, new causative CNVs, and the independent predictors of significant CNVs. Thirty-one CNVs (25 pathogenic and 6 likely pathogenic) in 28% (28/100) of cases were identified and 17 cases had 18 known syndromes. One case had two CNVs that presented two different syndromes: 3q29 microduplication syndrome and 1q43q44 microdeletion syndrome. Three rare non-recurrent CNVs encompassing dosage-sensitive genes were identified: deletion at 19q13.2 (*ATP1A3*), Xp11.4-p11.3 (*CASK*), and 6q25.3-q25.3 (*ARID1B*). The relative burden conveyed by each clinical feature accompanying unexplained ID/GDD-EP was evaluated by both univariate and multivariate analysis in 84 patients (28 patients with significant CNVs and 56 patients with non-significant CNVs). Upon seeking the association between significant CNVs and clinical findings of patients with unexplained ID/GDD-EP, we found that speech and language delay for those aged above 2 years (*P* = 0.025), facial malformations (*P* = 0.000), microcephaly (*P* = 0.014), congenital heart disease (*P* = 0.005), fair skin (*P* = 0.034), eye malformations (*P* = 0.037), and mega cisterna magna (*P* = 0.034) were associated with positive CNV tests. However, only eye malformations (*P* = 0.032) and facial malformations (*P* = 0.027) were independent predictors according to multivariate logistic regression. Their positive and negative predictive values were 65 and 80%, respectively.

The role of CNVs has been well-studied in children with EP (20–22) and ID/GDD (23, 24); however, only a few studies have focused on patients with ID/GDD-EP. The diagnostic yield of CNV tests in patients with unexplained EP ranges from 5 to 15% (21, 25, 26), while for individuals with unexplained ID/GDD, ASD, or multiple congenital anomalies, it is estimated to range from 15 to 20% (11). This study of pediatric patients with unexplained ID/GDD-EP revealed that a high proportion of our patients had clinically relevant CNVs. Twenty-eight probands (28%) had 25 pathogenic and 6 likely pathogenic CNVs. Fry et al. and Mullen et al. studied pediatric patients with genetic generalized epilepsy with ID, whereby they found the prevalence of pathogenic and likely pathogenic CNVs ranging from 8.8 to 28% (27, 28), respectively. Our finding is similar to that of Mullen et al. (28). Borlot et al. investigated adult patients with pediatric-onset ID and EP, whereby the prevalence of pathogenic and likely pathogenic CNVs was found to be 16.1% (10). Their study was based in Canada where there are multiple ethnicities. It involved 143 adults in which most of them had mild ID associated with focal or febrile seizures that started 1 year after birth. Our study involved 100 Chinese children in which most of them had severe ID associated with focal seizures that started within the first year of delivery. Our study revealed a higher diagnostic yield compared to that of Borlot et al. This can be explained by the fact that in most cases, patients with severe ID/GDD and EP end up dying early, when compared to those with mild ID/GDD and EP. We speculate that the study performed by Borlot et al. might have missed some cases with severe ID/GDD due to early deaths. However, the higher diagnostic yield in our study could also be due to ascertainment bias. Consequently, our findings support the usage of CNV testing in pediatric patients with unexplained ID/GDD-EP, as there is a high diagnostic yield which informs genetic counseling.

Eighteen known syndromes were diagnosed in 17 cases with pathogenic CNVs: Angelman syndrome for two cases, Prader-Willi syndrome for two cases, Lubs X-linked mental retardation syndrome for three cases, 16p11.2-p12.2 microdeletion syndrome for three cases, 1p36 microdeletion syndrome for two cases, 2q33.1 deletion syndrome/Glass syndrome for one case, 17p13.1 microdeletion syndrome for one case, 1q21.1 recurrent microdeletion syndrome for one case, 3q29 microduplication syndrome and 1q43q44 microdeletion syndrome for one case, and Wolf-Hirschhorn syndrome for one case. Borlot et al. found that 16 out of 23 adult patients with pathogenic or likely pathogenic CNVs and yet diagnosed with ID/GDD-EP had known syndromes (10). Therefore, our result along with that of Borlot et al. reinforces the supposition that a large number of patients with ID/GDD-EP might have CNVs which fall under certain microduplication or microdeletion syndrome.

One case (case number 20) had two CNVs that present two different syndromes:3q29 microduplication syndrome and 1q43q44 microdeletion syndrome (29). The 3q29 microduplication syndrome is characterized by mild-to-moderate ID/GDD, microcephaly, and minor dysmorphic features (30). The 1q43q44 microdeletion syndrome is characterized by ID/GDD, EP, microcephaly, anomalies of the corpus callosum, and facial dysmorphism (29). Our case presented with severe ID/GDD, EP, microcephaly, facial dysmorphism (hypertelorism, long and smooth philtrum, thin vermilion borders, and micrognathia), recurrent respiratory tract infections, and stereotypic movements. A *de novo* deletion at 1q44q44 spanning *HNRNPU* and *ZBTB18* has been described in cases with ID, EP, microcephaly, and hypogenesis of the corpus callosum (29, 31). This is similar to our patient's phenotype with the exception that she had no corpus callosum dysplasia. Our patient's CNV spans *HNRNPU* solely. The absence of *ZBTB18* in our patient's CNV could explain the lack of corpus callosum dysplasia. A *de novo* duplication at 3q29-q29 (880 Kb) spans *CEP19, PCYT1A, RNF168, TCTEX1D2*, and *TFRC*. However, none of the contained genes associate with ID/GDD or EP/both. Therefore, 1q43q44 microdeletion syndrome is likely to explain our patient's phenotype with few additional clinical features.

Three rare pathogenic CNVs of interest which span dosage-sensitive genes were identified. The first one is *de novo* deletion at 19q13.2 (333 Kb); this interval contains the *ATP1A3* gene. Mutations in the *ATP1A3* gene have been reported to be associated with alternating hemiplegia, as well as both ID/GDD and EP in a cohort of 34 patients (32). This coincides with our first patient's phenotype with an exception that he had hypotonia without hemiplegia; however, he will be continued to be monitored. The second CNV is deletion at Xp11.4-p11.3 (2.14 Mb); this interval contains the *CASK* gene. Mutation in this gene commonly affects females whereby they present with severe ID, microcephaly, and variable degrees of pontocerebellar hypoplasia (33). Additionally, some may have epileptic spasms, sensorineural hearing loss, eye anomalies, overall poor growth, dysmorphic features, including broad nasal bridge and tip, large ears, long philtrum, micrognathia, and hypertelorism (34). Our case number 4 presented with microcephaly, facial dysmorphism, and visual and hearing impairment, severe ID, late-onset epileptic spasms, and abnormal signals near the periventricular region. However, our case had no pontocerebellar hypoplasia. The third CNV is a deletion of unknown mode of inheritance at 6q25.3-q25.3 (240 Kb) spanning the *ARID1B* gene. This gene is associated with Coffin-Siris syndrome (ARID1B). Our case number 6 presented with moderate GDD, EP, facial dysmorphism (low frontal hairline, lateral sparse eyebrows, alternating ptosis, bulbous nasal tip, long and smooth philtrum, prominent upper lip, high palate, and mild retrognathia), abnormal fifth (pinky) finger, atrial septal defect, hearing loss, and enlarged cisterna magna and hence fits for Coffin-Siris syndrome. Consequently, we conclude that these three CNVs are implicated in ID/GDD-EP.

Other CNVs contain genes with unclear function, but they correlate with the patients' individual phenotypes and are expressed in the brain and, overall, present a lower threshold to variation according to their RVISs: duplication at 4q24-q24 (*CENPE*) for two cases, 10q21.2-q21.2 (*ANK3*), 14q32.31-q32.31 (*DYNC1H1*), 1p34.2-p34 (*SZT2*), and Xp21.3-p21.3 (*IL1RAPL1*). More studies are needed to prove their roles in ID/GDD-EP.

In this study, it was also found that congenital eye and facial malformations can independently predict the presence of significant CNVs among pediatric patients with unexplained ID/GDD-EP ([*P* = 0.032] and [*P* = 0.027], respectively). Their positive and negative predictive values were 65% and 80%, respectively. This is to say that 65% of the patients with these clinical features might have significant CNVs, whereas 80% of the patients lacking these clinical features might have non-significant CNVs. This is the first study that investigated the predictive values of the clinical features; hence, it remains non-conclusive. Studies involving larger sample sizes are invited. Preiksaitiene et al. studied a large cohort of patients with ID/GDD and found that there is a significant association between eye malformations and significant CNVs; however, they could not stand as independent predictors in their study (35). Our study marks the first time that congenital eye malformations are reported to be an independent predictor of the presence of significant CNVs. Caramaschi et al. studied patients with ID/GDD and found that facial malformation can be an independent predictor of presence of significant CNVs (14). The combination of the aforementioned studies and ours suggests that the presence of facial malformations in patients with either ID/GDD or ID/GDD-EP indicates the presence of pathogenic or likely pathogenic CNVs in 65% of all the cases.

In addition, speech and language delay for those aged above 2 years (*P* = 0.025), microcephaly (*P* = 0.014), congenital heart disease (*P* = 0.005), fair skin (*P* = 0.034), and mega cisterna magna (*P* = 0.034) showed significant associations; however, they could not stand as independent predictors in our cohort. This could be attributed by the usage of a small sample size. Nevertheless, they are promising indicators that need more studies with larger sample size. Shoukier et al. also found a significant association between significant CNVs and microcephaly in patients with ID/GDD (15). Positive association between significant CNVs and congenital heart disease in patients with ID/GDD has been reported (13, 36, 37). A chromosomal aberration can contribute to the etiology of speech and language delay (38, 39). Fair skin is commonly seen in patients with Prader-Willi syndrome and Angelman syndrome (40, 41), and it was also noticed in our patients with these diagnoses. Isolated mega cisterna magna can lead to impaired memory and speech (42). It has been reported to be associated with psychiatric conditions such as mania, autism, catatonic schizophrenia (43), and mild syndromic ID (44). This association points out its role in neurodevelopmental disorders. This is the first study that shows that there is a significant association between pathogenic or likely pathogenic CNVs with speech delay and mega cisterna magna. This could be due to our inclusion criteria of patients with exclusive unexplained ID/GDD-EP. Other studies involved patients with ID/GDD with or without EP. Lastly, we could not find any association between significant CNVs and seizure semiology or EEG findings.

Despite all aforementioned strengths, our study has some limitations. First, it involved a small sample size and was prone to information bias because it was a retrospective study. Second, we identified many CNVs whose pathogenic mechanisms could not be explained, since no gene function analysis was performed. Therefore, those CNVs require further functional evaluation and comparison with similar cases. Moreover, we identified two clinical markers for pediatric patients with ID/GDD-EP, but these findings need to be replicated in future studies involving large sample sizes. Lastly, different CNV detection methods were used whereby they have different sensitivities.

## Conclusion

Twenty-eight percent (28/100) of our cases had 25 pathogenic and 6 likely pathogenic CNVs, including 18 syndromes. However, the high rate (28%) of significant CNVs in our study could be due to ascertainment bias. We have identified 13 rare CNVs (8 novel and 5 reported in the literature) in which three of them span dosage-sensitive genes as follows: 19q13.2 deletion (*ATP1A3*), Xp11.4-p11.3 deletion (*CASK*), and 6q25.3-q25.3 deletion (*ARID1B*). Furthermore, we found two independent clinical markers with 65% positive predictive value: congenital eye and facial malformations ([*P* = 0.032] and [*P* = 0.027]), respectively. These clinical markers help clinicians understand which patients can benefit from the CNV testing and which will not, thus helping patients to avoid unnecessary and expensive tests, especially for resource-limited countries.

## Author contributions

MK and JX share the primary authorship for this manuscript. MK and JX participated in the entire process of the project, analyzed all the data, and wrote the manuscript. LW and LY analyzed patients' data. FH and CC prepared tables. NP, HD, WZ, and AA analyzed cytogenetic results according to ACMG guidelines. FY and JP are the corresponding authors, who initiated the study and supervised each and every step of this study. All of the authors have reviewed the manuscript and agreed to be accountable for all aspects of work ensuring integrity and accuracy.

### Conflict of interest statement

The authors declare that the research was conducted in the absence of any commercial or financial relationships that could be construed as a potential conflict of interest.

## References

[1] van Bokhoven H Genetic and epigenetic networks in intellectual disabilities. Annu Rev Genet. (2011) 45:81–104. 10.1146/annurev-genet-110410-132512 21910631

[2] Shevell M Ashwal S Donley D Practice parameter: evaluation of the child with global developmental delay: report of the Quality Standards Subcommittee of the American Academy of Neurology and The Practice Committee of the Child Neurology Society Practice parameter: Evaluation of t. Am Acad Neurol. (2003) 60:367–80. 10.1212/01.WNL.0000031431.81555.16 12578916

[3] Srour M Shevell M. Genetics and the investigation of developmental delay/intellectual disability. Arch Dis Child (2014) 99:386–9. 10.1136/archdischild-2013-304063 24344174

[4] Fisher RS Acevedo C Arzimanoglou A Bogacz A Cross JH Elger CE. ILAE Official Report: a practical clinical definition of epilepsy. Epilepsia (2014) 55:475–82. 10.1111/epi.12550 24730690

[5] Oeseburg B Dijkstra GJ Groothoff JW Reijneveld SA Jansen DEMC. Prevalence of chronic health conditions in children with intellectual disability: a systematic literature review. Intellect Dev Disabil (2011) 49:59–85. 10.1352/1934-9556-49.2.59 21446871

[6] Robertson J Hatton C Emerson E Baines S. Prevalence of epilepsy among people with intellectual disabilities: a systematic review. Seizure (2015) 29:46–62. 10.1016/j.seizure.2015.03.016 26076844

[7] Robertson J Hatton C Emerson E Baines S. Mortality in people with intellectual disabilities and epilepsy: a systematic review. Seizure (2015) 29:123–33. 10.1016/j.seizure.2015.04.004 26076855

[8] Camfield C Camfield P. Preventable and unpreventable causes of childhood-onset epilepsy plus mental retardation. Pediatrics (2007) 120:e52–5. 10.1542/peds.2006-3290 17606549

[9] Schaaf CP Wiszniewska J Beaudet AL. Copy number and SNP arrays in clinical diagnostics. Annu Rev Genomics Hum Genet. (2011) 12:25–51. 10.1146/annurev-genom-092010-110715 21801020

[10] Borlot F Regan BM Bassett AS Stavropoulos DJ Andrade DM. Prevalence of pathogenic copy number variation in adults with pediatric-onset epilepsy and intellectual disability. JAMA Neurol. (2017) 74:1301–11. 10.1001/jamaneurol.2017.1775 28846756 PMC5710585

[11] Miller DT Adam MP Aradhya S Biesecker LG Brothman AR Carter NP Consensus statement: chromosomal microarray is a first-tier clinical diagnostic test for individuals with developmental disabilities or congenital anomalies. Am J Hum Genet. (2010) 86:749–64. 10.1016/j.ajhg.2010.04.006 20466091 PMC2869000

[12] Cappuccio G Vitiello F Casertano A Fontana P Genesio R Bruzzese D. New insights in the interpretation of array-CGH: autism spectrum disorder and positive family history for intellectual disability predict the detection of pathogenic variants. Ital J Pediatr. (2016) 42:1–11. 10.1186/s13052-016-0246-7 27072107 PMC4830019

[13] Shoukier M Klein N Auber B Wickert J Schröder J Zoll B. Array CGH in patients with developmental delay or intellectual disability: are there phenotypic clues to pathogenic copy number variants? Clin Genet. (2013) 83:53–65. 10.1111/j.1399-0004.2012.01850.x 22283495

[14] Maini I Ivanovski I Djuric O Caraffi SG Errichiello E Marinelli M Prematurity, ventricular septal defect and dysmorphisms are independent predictors of pathogenic copy number variants: a retrospective study on array-CGH results and phenotypical features of 293 children with neurodevelopmental disorders and/or multiple c. Ital J Pediatr. (2018) 44:1–13. 10.1186/s13052-018-0467-z 29523172 PMC5845186

[15] Caramaschi E Stanghellini I Magini P Giuffrida M Scullin S Giuva T. Predictive diagnostic value for the clinical features accompanying intellectual disability in children with pathogenic copy number variations: a multivariate analysis. Ital J Pediatr. (2014) 40:39. 10.1186/1824-7288-40-39 24775911 PMC4014206

[16] Shevell M. Global developmental delay and mental retardation or intellectual disability: conceptualization, evaluation, and etiology. Pediatr Clin North Am. (2008) 55:1071–84. 10.1016/j.pcl.2008.07.010 18929052

[17] Guo H Peng Y Hu Z Li Y Xun G Ou J. Genome-wide copy number variation analysis in a Chinese autism spectrum disorder cohort. Sci Rep. (2017) 7:1–9. 10.1038/srep44155 28281572 PMC5345089

[18] Kearney HM Thorland EC Brown KK Quintero-Rivera F South ST. American College of Medical Genetics standards and guidelines for interpretation and reporting of postnatal constitutional copy number variants. Genet Med. (2011) 13:680–5. 10.1097/GIM.0b013e3182217a3a 21681106

[19] Petrovski S Wang Q Heinzen EL Allen AS Goldstein DB. Genic intolerance to functional variation and the interpretation of personal genomes. PLoS Genet. (2013) 9:e1003709. 10.1371/journal.pgen.1003709 23990802 PMC3749936

[20] Ma Y Chen C Wang Y Wu L He F Chen C. Analysis copy number variation of Chinese children in early-onset epileptic encephalopathies with unknown cause. Clin Genet. (2016) 90:428–36. 10.1111/cge.12768 26925868

[21] Olson H Shen Y Avallone J Sheidley BR Pinsky R Bergin AM. Copy number variation plays an important role in clinical epilepsy. Ann Neurol. (2014) 75:943–58. 10.1002/ana.24178 24811917 PMC4487364

[22] Mefford HC Yendle SC Hsu C Cook J Geraghty E McMahon JM. Rare copy number variants are an important cause of epileptic encephalopathies. Ann Neurol. (2011) 70:974–85. 10.1002/ana.22645 22190369 PMC3245646

[23] Roselló M Martínez F Monfort S Mayo S Oltra S Orellana C. Phenotype profiling of patients with intellectual disability and copy number variations. Eur J Paediatr Neurol. (2014) 18:558–66. 10.1016/j.ejpn.2014.04.010 24815074

[24] Coutton C Dieterich K Satre V Vieville G Amblard F David M. Array-CGH in children with mild intellectual disability: a population-based study. Eur J Pediatr. (2014) 174:75–83. 10.1007/s00431-014-2367-6 24985125

[25] Boutry-Kryza N Labalme A Ville D de Bellescize J Touraine R Prieur F. Molecular characterization of a cohort of 73 patients with infantile spasms syndrome. Eur J Med Genet. (2015) 58:51–8. 10.1016/j.ejmg.2014.11.007 25497044

[26] Allen NM Conroy J Shahwan A Ennis S Lynch B Lynch SA. Chromosomal microarray in unexplained severe early onset epilepsy - A single centre cohort. Eur J Paediatr Neurol. (2015) 19:390–4. 10.1016/j.ejpn.2015.03.010 25920948

[27] Fry AE Rees E Thompson R Mantripragada K Blake P Jones G. Pathogenic copy number variants and SCN1A mutations in patients with intellectual disability and childhood-onset epilepsy. BMC Med Genet. (2016) 17:34. 10.1186/s12881-016-0294-2 27113213 PMC4845474

[28] Mullen SA Carvill GL Bellows S Bayly MA Berkovic SF Dibbens LM. Copy number variants are frequent in genetic generalized epilepsy with intellectual disability. Neurology (2013) 81:1507–14. 10.1212/WNL.0b013e3182a95829 24068782 PMC3888172

[29] Depienne C Nava C Keren B Heide S Rastetter A Passemard S. Genetic and phenotypic dissection of 1q43q44 microdeletion syndrome and neurodevelopmental phenotypes associated with mutations in ZBTB18 and HNRNPU. Hum Genet. (2017) 136:463–79. 10.1007/s00439-017-1772-0 28283832 PMC5360844

[30] Lisi EC Hamosh A Doheny KF Squibb E Jackson B Galczynski R 3q29 Interstitial microduplication: a new syndrome in a three-generation family. Am J Med Genet Part A (2008) 146:601–9. 10.1002/ajmg.a.32190 18241066

[31] Westphal DS Andres S Beitzel KI Makowski C Meitinger T Hoefele J. Identification of a *de novo* microdeletion 1q44 in a patient with hypogenesis of the corpus callosum, seizures and microcephaly – A case report. Gene (2017) 616:41–4. 10.1016/j.gene.2017.03.025 28336463

[32] Panagiotakaki E De Grandis E Stagnaro M Heinzen EL Fons C Sisodiya S. Clinical profile of patients with ATP1A3 mutations in Alternating Hemiplegia of Childhood - A study of 155 patients. Orphanet J Rare Dis. (2015) 10:123. 10.1186/s13023-015-0335-5 26410222 PMC4583741

[33] Moog U Bierhals T Brand K Bautsch J Biskup S Brune T. Phenotypic and molecular insights into CASK-related disorders in males. Orphanet J Rare Dis. (2015) 10:44. 10.1186/s13023-015-0256-3 25886057 PMC4449965

[34] Nakajiri T Kobayashi K Okamoto N Oka M Miya F Kosaki K. Late-onset epileptic spasms in a female patient with a CASK mutation. Brain Dev. (2015) 37:919–23. 10.1016/j.braindev.2015.02.007 25765806

[35] Preiksaitiene E Molyte A Kasnauskiene J Ciuladaite Z Utkus A Patsalis PC. Considering specific clinical features as evidence of pathogenic copy number variants. J Appl Genet. (2014) 55:189–96. 10.1007/s13353-014-0197-x 24535828

[36] Lu X-Y Phung MT Shaw CA Pham K Neil SE Patel A. Genomic imbalances in neonates with birth defects: high detection rates by using chromosomal microarray analysis. Pediatrics (2008) 122:1310–8. 10.1542/peds.2008-0297 19047251 PMC2795566

[37] Cooper GM Coe BP Girirajan S Rosenfeld JA Vu TH Baker C. A copy number variation morbidity map of developmental delay. Nat Genet. (2011) 43:838–46. 10.1038/ng.909 21841781 PMC3171215

[38] Utami KH Hillmer AM Aksoy I Chew EGY Teo ASM Zhang Z. Detection of chromosomal breakpoints in patients with developmental delay and speech disorders. PLoS ONE (2014) 9:e90852. 10.1371/journal.pone.0090852 24603971 PMC3946304

[39] Cao Q Peng Y Ge JUN Zhang Y Zhu J Zhao L. A novel 5p15.33-14.1 deletion and 4q34.24-35.2 duplication in a patient with mental retardation, dysmorphic features and severe speech delay. J Genet. (2014) 93:159–62. 10.1007/s12041-014-0318-7 24840832

[40] Angulo MA Butler MG Cataletto ME. Prader-Willi syndrome: a review of clinical, genetic, and endocrine findings. J Endocrinol Invest. (2015) 38:1249–63. 10.1007/s40618-015-0312-9 26062517 PMC4630255

[41] Wheeler AC Sacco P Cabo R. Unmet clinical needs and burden in Angelman syndrome: a review of the literature. Orphanet J Rare Dis. (2017) 12:1–17. 10.1186/s13023-017-0716-z 29037196 PMC5644259

[42] Zimmer EZ Lowenstein L Bronshtein M Goldsher D Aharon-Peretz J. Clinical significance of isolated mega cisterna magna. Arch Gynecol Obstet. (2007) 276:487–90. 10.1007/s00404-007-0369-6 17453222

[43] Pandurangi S Pandurangi A Matkar A Shetty N Patil P. Psychiatric manifestations associated with mega cisterna magna. J Neuropsychiatry Clin Neurosci. (2014) 26:169–71. 10.1176/appi.neuropsych.13040097 24763763

[44] Konca Ç Caliskan B Tas MA. A case with mega cisterna magna renal and ear anomalies: is this a new syndrome? Case Rep Med. (2013) 2013:3–7. 10.1155/2013/149656 23762068 PMC3671267

