# Supplementary material for: Rare Copy Number Variations and Predictors in Children With Intellectual Disability and Epilepsy
Source: Front Neurol. 2018 Nov 19;9:947. doi: 10.3389/fneur.2018.00947 (PMC6252327; doi:10.3389/fneur.2018.00947)
Supplement: Supplementary file 1 [file Table_1.DOCX]

**Supplementary Table 1**

**Copy number variation test results together with clinical features of the patients with pathogenic or likely pathogenic CNV.**

| **Patient No/Sex** | **ID/**  **GDD onset age**  **(months)** | **EP onset age**  **(months)** | **Seizure type** | **ID/GDD category** | **Clinical features** | **CNV** | **CNV**  **inheritance**  **validation method** | **Genomic coordinates**  **(NCBI 37/hg19)** | **Associated genetic syndrome** | **Interpretation** |
| --- | --- | --- | --- | --- | --- | --- | --- | --- | --- | --- |
| 1/M | 3 | 7 | Tonic | Moderate | Motor development delay, speech delay, hearing impairment, facial dysmophism (frontal bossing and low set ears), blue nevus, hypotonia, clonus, positive Babinski sign, brain atrophy and patent foramen ovale. | *De novo* 19q13.2 del (333 Kb) | aCGH+SNP Array | Chr19:  42428679-42762051 | NA | P |
| 2/M | 24 | 48 | Tonic | Severe | Speech delay, microcephaly, skeletal malformations, strabismus, unsteady gait, dysmorphic features (including deviated mouth, epicanthus, downslanted palpebral fissures low set ears, button nose, prominent nasal bridge, and skin pigmentation), hypotonia, abnormal behavior, sleep disturbances, hand apraxia, stereotypic movement and mega cisterna magna. | Unknown inheritance 17p13.1-p13.1 del (380 Kb) | NA | Chr17:  6861627-7241627 | 17p13.1 microdeletion syndrome  (OMIM 613776) | P |
| 3/F | 3 | 37 | Tonic | Severe | Speech delay, spluttering, abnormal skin, fine motor difference, dysmetria, dysarthria, and recurrent infections. | Unknown inheritance 10q11.23q22.1 del (21 Mb) | NA | Chr10:  51795395-73315940 | NA | P |
| 4/F | 3 | 48 | Myoclonus, spasm | Severe | Microcephaly, facial dysmorphism, stunted growth, visual and hearing impairment, late-onset epileptic spasms, patent foramen ovale, stereotypic movement including clapping hands, abnormal signals near the periventricular region. | Unknown inheritance Xp11.4-p11.3  del (2.14 Mb) | NA | ChrX:  41293257-43433257 | NA | P |
| 5/M | 10 | 59 | Focal | Severe | Dysmorphic features, microcephaly, (frontal bossing, deep-set eyes, and bulbous nose), grumpy and aggressive behavior, cryptorchidism, cachexia and dry skin. | *De novo*1q21.1-q21.2 del (3.99 Mb) | CNV-seq | Chr1:  143896003-147888300 | 1q21.1 recurrent microdeletion  syndrome  (OMIM 612474) | P |
| 6/F | 3 | 94 | Tonic | Moderate | Facial dysmorphism (low frontal hairline, lateral sparse eyebrows, alternating ptosis, bulbous nasal tip, long and smooth philtrum, prominent upper lip, high palate, and mild retrognathia), microcephaly, abnormal fifth pink fingers, atrial septal defect, hearing loss and enlarged cisterna magna. | Unknown inheritance  6q25.3-q25.3  del (240 Kb) | NA | Chr6:  156994744-157234744 | NA | P |
| 7/F | 3 | 40 | Myoclonus | Severe | Severe limitations in speech and language, fair skin changes, ventricular septal defect, dysmorphic features (curly hair, small ears, strabismus, high arched palate, micrognathia), hypotonia, ataxic gait, visual impairment and stereotypic movement. | Unknown inheritance 15q11.2-q13.1  del (4.92 Mb) | NA | Chr15:  23609854-28526207 | Angelman syndrome  (OMIM 105830) | P |
| 8/F | 3 | 5 | Focal | Moderate | Diminished fetal activity, muscular hypotonia, short stature, microcephaly, small hands and feet, fair skin changes, facial dysmorphism (high prominent forehead and micrognathia) and patent foramen ovale. | Unknown inheritance 15q11.2-q13.1  del (4.93 Mb) | NA | Chr15:  23609854-28536207 | Prader-Will syndrome  (OMIM 176270) | P |
| 9/M | 3 | 5 | Focal | Severe | Treatable seizures, mongolian macula, microcephaly, cleft palate, mild facial dysmorphism and syndactyly. | Unknown inheritance 16p11.2-p11.2  del (550 Kb) | NA | Chr16: 29661922-30211922 | 16p11.2-p12.2 microdeletion syndrome  (OMIM 613604) | P |
| 10/M | 18 | 72 | Focal | Moderate | Low birth weight, delayed psychomotor development, attention deficit hyperactivity disorder, facial dysmorphism (spares teeth, protruding ears and deviated mouth). | *De novo*  10q23.31-q24.33 dup (14.82Mb) | CNV-seq | Chr10:  90175157-104995157 | NA | P |
| 11/M | 6 | 5 | Focal | Mild | Social withdrawal and attention deficit hyperactivity disorder. | *De novo*  4q24-q24  dup (150 Kb) | qPCR | Chr4:  104007280-104157280 | NA | LP |
| 12/M | 24 | 142 | Tonic | Moderate | Autism (absent verbal communication and stereotypic movement) and facial dysmorphism (epicanthic folds, short nose with a broad raised tip, and hypertelorism) | Unknown inheritance  1p34.2-p34.2  dup (160 Kb) | NA | Chr1:  43823083-43983083 | NA | LP |
|  |  |  |  |  |  | Unknown inheritance  4q24-q24  dup (170 Kb) | NA | Chr4:  103977280-104147280 | NA | LP |
|  |  |  |  |  |  | Unknown inheritance 10q21.2-q21.2  dup (180 Kb) | NA | Chr10:  61747807-61927807 | NA | LP |
| 13/M | 5 | 65 | Tonic-clonic | Severe | Speech delay, hypotonia and hypothyroidism. | Unknown inheritance  Xq28-q28  dup (340 Kb) | NA | ChrX:  153232100-153572100 | Lubs X-linked mental retardation syndrome(300260) | P |
| 14/M | 3 | 6 | Tonic | Severe | Speech delay, obesity, mild facial dysmorphism (protruding ears and sparse teeth) and arachnoid cyst. | Unknown inheritance  Xp21.3-p21.3  dup (110 Kb) | NA | ChrX: 28993675-29103675 | NA | LP |
| 15/M | 3 | 18 | Tonic | Severe | Speech delay, microcephaly, small genitalia, short stature, truncal obesity, dysmorphic facies, strabismus, hypertelorism and syndactyly. | Unknown inheritance 15q11.2-q13.1  del (5.15 Mb) | NA | Chr15: 23609854- 28756207 | Prader-Willi syndrome (OMIM 176270) | P |
| 16/M | 48 | 24 | Tonic | Moderate | Speech delay, mild dysmorphic features (including hypotelorism, flat nasal bridge, and short philtrum). | Unknown inheritance  Xq28-q28  dup (210 Kb) | NA | ChrX: 153362100- 153572100 | Lubs X-linked mental retardation syndrome(30060) | P |
| 17/F | 3 | 0 | Focal, spasm | Severe | Speech delay, craniofacial malformations (including deep set eyes, straight eyebrows, midface hypoplasia, broad flat nose, long philtrum, abnormal location of the pinna, microcephaly and cerebral atrophy), enamel dysplasia, skin abnormality (including hirsutism and abnormal palms), short fifth fingers, hypotonia and positive Barbinski sign. | Unknown inheritance 1p36.33-p36.33  del (1.10 Mb) | NA | Chr1:  746369- 1846369 | 1p36 microdeletion syndrome  (OMIM 607872 ) | P |
| 18/M | 3 | 4 | Focal | Moderate | Speech delay, microcephaly, dysmorphic features including flat bossing skull, flat nasal bridge, micrognathia, and scrotal hemangioma. | Unknown inheritance 16p11.2-p11.2 del (640 Kb) | NA | Chr16: 29571922- 30211922 | 16p11.2-p12.2 microdeletion syndrome  (OMIM613604) | P |
| 19/F | 3 | 53 | Focal, tonic | Severe | Speech delay, scoliosis and stereotypic movement. | Unknown inheritance 14q32.31-q32.31 dup (140 Kb) | NA | Chr14:  102405001- 102545001 | NA | LP |
| 20/F | 3 | 10 | Focal | Severe | Facial dysmorphism (hypertelorism, long and smooth philtrum, thin vermilion borders, and micrognathia), microcephaly, recurrent respiratory tract infections and stereotypic movements. | *De novo*  1q44q44  del (4.6Mb) | CNV-seq | Chr1: 244307212- 248903211 | 1q43q44 microdeletion syndrome[[76](#_ENREF_76)] | P |
|  |  |  |  |  |  | *De novo*  3q29-q29 dup (880Kb) | CNV-seq | Chr3: 195732252- 196612252 | 3q29 microduplication syndrome  (OMIM 611936) | P |
| 21/M | 8 | 14 | Tonic | Moderate | Speech delay, hypotonia, mild dysmorphic features (including prominent forehead, microphthalmia, and flat nasal bridge) and stereotypic movements. | Unknown inheritance  Xq28-q28  dup (210 Kb) | NA | ChrX: 153362100- 153572100 | Lubs X-linked mental retardation syndrome(300260) | P |
| 22/M | 3 | 18 | Tonic | Moderate | Speech delay, sparse hair, aggressive behaviors, dysmorphic features (including low set ears, ptosis, bulbous nose, visual and hearing impairment, and strabismus) and brachydactyly. | Unknown inheritance  8p21.2-p21 del (10.8Mb) | NA | Chr8: 23526855- 34326855 | NA | P |
| 23/F | 3 | 36 | Tonic | Severe | Speech delay, microcephaly, fair skin changes, dysmorphic facies (including micrognathia, strabismus and high arched palate), hypotonia, stereotypic movement, excitable personality and inappropriately happy affect. | Unknown inheritance 15q11.2-q13.1 del (6.01 Mb) | NA | Chr15: 22751194- 28756207 | Angelman syndrome  (OMIM 105830) | P |
| 24/M | 3 | 30 | Tonic, spams | Moderate | Speech delay, brachydactyly and protruding ears, dysarthria, ataxic gait and dyslexia. | *De novo*  8p23.3-p23.2  del (5.57 Mb) | CNV-seq | Chr8:  155001- 5725001 | NA | P |
| 25/M | 12 | 5 | Tonic | Moderate | Dysmorphic facies (including flat nasal bridge) and treatable seizures. | *De novo*  16p11.2-p11.2  del (640 Kb) | CNV-seq | Chr16: 29571922- 30211922 | 16p11.2-p12.2 microdeletion syndrome (OMIM 613604) | P |
| 26/F | 48 | 12 | Tonic | Moderate | Dysmorphic facies (including deeply set eye and micrognathia), delayed cranial suture closure, hypotonia, and subcutaneous nodules. | Unknown inheritance 1p36.32-p36.23 del (5.3 Mb) | NA | Chr1:3417960-8774451 | 1p36 microdeletion syndrome  (OMIM 607872 ) | P |
| 27/F | 3 | 72 | Focal | Severe | Speech delay, dysmorphic features (including sparse hair, high palate, small mouth, and epicanthus), short stature and destructive behavior. | *De novo*  2q33.1-q34 del (8.9 Mb) | De novo/  FISH | Chr2:202781244-211695915 | 2q33.1 deletion syndrome | P |
| 28/F | 6 | 7 | Focal | Severe | Low birth weight: hypotonia: dysmorphic features (including microcephaly, epicanthus, short nose and philtrum, and everted lower eyelids): scoliosis: sacral sinus: ear deformity: hyperreflexia and corpus callosum dysplasia. | Unknown inheritance  4p16.3-p16.1  del (7.3 Mb) | NA | Chr4:  68345-7109830 | Wolf-Hirschhorn syndrome  (OMIM 194190) | P |

M=male; F=female; CNV=copy number variation; P=pathogenic; LP=likely pathogenic; Del=deletion; Dup=duplication; ID=intellectual disability; GDD=global developmental delay; EP=epilepsy; NA=not applicable; OMIM=Online Mendelian Inheritance in Man; NA=not applicable
